# 

**Published:** 1876-11

**Authors:** 


### Association of the Alumni and Officers of the Medical Department of the University of Buffalo.

The third annual meeting of this Association will be held at the College Building, on Commencement day, Tuesday, February 20th, and an interesting re-union is anticipated. The committee of arrangements have not yet completed their programme, but we are permitted to state that at the meeting in the afternoon some interesting topics connected with medical practice, will be presented for discussion. In the evening at the conclusion of the commencement exercises Prof. F. H. Hamilton, of New York, will deliver the address to the Alumni. Arrangements are being made for a supper at the conclusion of the evening exercises.

The executive committee do not desire to exclude volunteer papers from the meeting, but request that their titles and probable length be sent to the Secretary, Dr. E. N. Brush, prior to the twentieth of February.

---

A new exchange, THE TOLEDO MEDICAL AND SURGICAL JOURNAL, Jonathan Priest, M. D., editor, is announced and will be welcomed to our list.——THE CLINIC (Cincinnati) will after January 1st, pass under the control of Dr. Roberts, Bartholow.——Among the awards at the Centennial Exhibition we notice that Messrs. Warner & Co., of Philadelphia, received the medal of award over all other competitors for sugar coated pills.

### Books and Pamphlets Received.

Chemistry: General, Medical and Pharmaceutical, Including the Chemistry of the U. S. Pharmacopœia. A Manual of the General Principles of the Science, cnd their applications in Medicine and Pharmacy. By John Attfield, Ph. D., F. C. S. Philadelphia: Henry C. Lea, 1876. Buffalo: T. Butler & Son.

The Use and Value of Arsenic in the Treatment of Skin Diseases. By L. Duncan Bulkley, A M., M. D. New York: D. Appleton & Co., 1876.

Public Libraries in the United States: Their History, Condition and Management. Special Report. Department of the Interior, Washington, 1876.

The Operation for Stone as observed in some of the London Hospitals: Together with Report of Cases from Private Practice. By A. Van Derveer, M. D. Reprint from the Archives of Clinical Surgery, October, 1876.

### Contents of November Number.

### ORIGINAL.

ART. I.—The Rational Treatment of Epilepsy. By Reuben A. Vance, M. D., .......... 123
ART. II.—Sarcoma of the Dura Mater. By E. N. Brush, M. D., ...... 126

### CORRESPONDENCE.

THE BUFFALO

**Medical and Surgical Journal**

PUBLISHED MONTHLY,

**Containing Original Articles, Reports of Medical Societies and Hospitals, Editorial, Reviews, Correspondence, Army News, etc., etc.; including the usual variety of Medical Periodical Publications. Specimen copies sent on application.**

Address Buffalo Medical & Surgical Journal, Buffalo, N. Y.

TERMS OF SUBSCRIPTION, $3.00 PER YEAR, IN ADVANCE.

